# Metatranscriptome-based strategy reveals the existence of novel mycoviruses in the plant pathogenic fungus *Fusarium oxysporum* f. sp. *cubense*

**DOI:** 10.3389/fmicb.2023.1193714

**Published:** 2023-05-18

**Authors:** Yiting Ye, Yingying Liu, Yifei Zhang, Xin Wang, Huaping Li, Pengfei Li

**Affiliations:** Guangdong Province Key Laboratory of Microbial Signals and Disease Control, College of Plant Protection, South China Agricultural University, Guangzhou, China

**Keywords:** *Fusarium oxysporum* f. sp. *cubense*, mycovirus, metatranscriptome, virus diversity, biocontrol

## Abstract

*Fusarium oxysporum* f. sp. *cubense* (Foc) is a devastating plant pathogen that caused a great financial loss in the banana’s source area. Metatranscriptomic analysis was used to determine the diversity of mycoviruses in 246 isolates of *F. oxysporum* f. sp. *cubense*. Partial or nearly complete genomes of 20 mycoviruses were obtained by BLASTp analysis of RNA sequences using the NCBI database. These 20 viruses were grouped into five distinct lineages, namely *Botourmiaviridae*, *Endornaviridae*, *Mitoviridae*, *Mymonaviridae*, *Partitiviridae*, and two non-classified mycoviruses lineages. To date, there is no report of the presence of mycoviruses in this pathogen. In this study, we demonstrate the presence of mycoviruses isolated from Foc. These findings enhance our overall knowledge of viral diversity and taxonomy in Foc. Further characterization of these mycoviruses is warranted, especially in terms of exploring these novel mycoviruses for innovative biocontrol of banana Fusarium wilt disease.

## Introduction

Bananas are cultivated in more than 120 countries and on approximately 11 million ha. In 2021, the global banana output reached 170.3 million tons, which is an increase of 3.6 million tons from 2020 (FAOSTAT, 2023). Banana Fusarium wilt, a devastating disease caused by the soil-borne, root-infecting fungus *Fusarium oxysporum* f. sp. *cubense* (Foc), is affecting global banana production. Based on the pathogenicity of banana cultivars, Foc is divided into three races, including Foc race 1 (Foc1), Foc race 2 (Foc2), and Foc race 4 (Foc4; Thangavelu, 2021). Foc 1 and Foc 4 are the most widespread physiological races in China, of which Foc 4 has been reported in almost all banana cultivation areas in China (Poon et al., 2020). According to geographical characteristics and temperature adaptation profiles, Foc race 4 is further subdivided into Tropical Race 4 (TR4) and Subtropical Race 4 (ST4; Ploetz, 2015). Banana Fusarium wilt has been known to be difficult to control because infection occurs through complex underground interactions between the fungus, the plant, and the soil microorganisms at the root-soil interface. Even after decades of research, there are few effective treatments for Fusarium wilt disease in bananas (Ploetz, 2015). Foc-resistant banana varieties are the most effective strategy for reducing Fusarium wilt impacts (Viljoen et al., 2020; Zorrilla-Fontanesi et al., 2020). However, conventional breeding is a time-consuming process that can take at least 15 years (Tenkouano et al., 2011). Therefore, biological control of banana Fusarium wilt disease is a promising strategy.

Mycoviruses are viruses that replicate in fungi and have been found in fungal kingdoms. While most mycoviruses are latent infections having no apparent effect on the fungal host, a few have major effects on host growth, development, and reproduction. Several mycoviruses have been identified that reduce the virulence of fungal plant pathogens, the most notable being Cryphonectria hypovirus 1 (CHV1), which reduces the virulence of *Cryphonectria parasitica*, the causal agent of chestnut blight (Ghabrial et al., 2015; Zhang and Nuss, 2016; Rigling and Prospero, 2018). Rosellinia necatrix megabirnavirus 1 (RnMBV1) in *Rosellinia necatrix* has been found to have significant potential to control apple white root rot disease (Kondo et al., 2013). Recently, a fungal DNA virus, Sclerotinia sclerotiorum hypovirulence-associated DNA virus 1 (SsHADV-1), has been shown to have the potential to control *Sclerotinia* disease (Liu et al., 2016). Furthermore, spraying SsHADV-1 infected strain DT-8 in the early flowering phase was reported to reduce rapeseed stem rot disease severity by 67.6% and improve yield by 14.9% (Zhang H. et al., 2020). Given the numerous examples of mycoviruses capable of attenuating the virulence of fungal pathogens, mycoviruses are promising tools for developing biological control strategies to limit the impact of fungi on crop productivity.

Mycoviruses are generally classified by the host, genome structure, and viral particle shape. The majority of known mycoviruses possess linear double-stranded RNA (dsRNA) or positive-sense (+) single-stranded RNA (ssRNA) genomes. However, a few mycoviruses with linear negative-sense (−) ssRNA and single-stranded DNA (ssDNA) genomes have also been reported (Myers and James, 2022). In recent decades, the variety of mycoviruses has increased massively. Next-generation sequencing (NGS) is a popular method for exploring mycoviral diversity, including sequencing total RNA depleted of ribosomal RNA or small RNA. Many novel viruses have been discovered with the help of NGS. For instance, 68 partial or nearly complete genome segments have been identified in the metatranscriptomes of three major fungal pathogens of rice (He et al., 2022). The metagenomic approach was used to comprehensively characterize all mycoviruses in an international collection of the brown rot pathogen *Monilinia fructicola* (De Miccolis Angelini et al., 2022). Based on the International Committee on Taxonomy of Viruses (ICTV) Taxonomy Report and ICTV Master Species List 2021.v3 (https://ictv.global/msl, accessed on 5 January 2023), there are 26 families containing over 200 mycovirus species. With the development and widespread use of RNA deep sequencing techniques, the diversity of mycoviruses in *Fusarium* species is constantly increasing. Mycoviruses have been described in many *Fusarium* species, such as *F. asiaticum* (Li et al., 2019), *F. andiyazi* (Yao et al., 2020), *F. boothii* (Mizutani et al., 2018), *F. circinatum* (Martínez-Álvarez et al., 2014), *F. coeruleum* (Osaki et al., 2015), *F. globosum* (Sharma et al., 2018), *F. graminearum* (Chu et al., 2002, 2004; Li et al., 2016; Zhang X. et al., 2020), *F. incarnatum* (Zhang et al., 2019), *F. langsethiae* (Li et al., 2017), *F. oxysporum* f. sp. *dianthi* (Lemus-Minor et al., 2015), *F. poae* (Osaki et al., 2016), *F. pseudograminearum* (Zhang et al., 2018), *F. sacchari* (Yao et al., 2020), *F. solani* (Osaki et al., 2015), *F. virguliforme* (Marvelli et al., 2014), and so on.

The majority of *Fusarium* mycoviruses establish latent infections, but some mycoviruses, such as Fusarium graminearum virus 1 (FgV1; Paudel et al., 2022), Fusarium graminearum virus-ch9 (FgV-ch9; Sharma et al., 2018), Fusarium graminearum hypovirus 2 (FgHV2; Li et al., 2015), and Fusarium oxysporum f. sp. dianthi mycovirus 1 (FodV1; Lemus-Minor et al., 2018), cause hypovirulence. However, no mycoviruses have been reported in *Fusarium oxysporum* f. sp. *cubense* (Foc), a devastating plant fungal pathogen. To fill this gap, we screened and cultured 246 strains isolated from southern and southwestern China (Fujian, Guangdong, Guangxi, Hainan, and Yunnan provinces) for metatranscriptome sequencing to investigate mycovirus diversity in Foc. This information provides insight into the diversity and taxonomy of mycoviruses of Foc.

## Materials and methods

### Isolates and growth conditions

Foc was isolated and purified from diseased banana plants in Fujian, Guangdong, Guangxi, Hainan, and Yunnan provinces, China. The fungal stock cultures were maintained in a final concentration of 25% (v/v) glycerol at −80°C. All isolates were cultured on potato dextrose agar (PDA: potato 200 g/L, agar 15 g/L, and dextrose 20 g/L) at 28°C in darkness.

### Total RNA extraction and purification

The total RNA of each strain was extracted from 0.5 g of fungal mycelium by using a Plant RNA Extraction Kit (Promega Code: LS1040, Beijing). The total RNA was stored at −80°C until used. 246 strains were divided into two groups, group 1 of 146 and group 2 of 100. Approximately 2 ng of RNA was taken from each sample and mixed. A pooled RNA sample was sent to Shanghai Biotechnology Corporation (China) for RNA sequencing (RNA-seq).

### RNA sequencing and sequence analysis

Ribosomal RNA depletion, library preparations, and Illumina sequencing were performed by Shanghai Biotechnology Corporation (China). Zymo-Seq RiboFree Total RNA Library Prep Kit (Zymo Research, USA) was used for ribosomal RNA depletion and library preparations. Illumina sequencing (Illumina NovaSeq 6000) was carried out by Shanghai Biotechnology Corporation (China).The filtered data was spliced together from scratch, and the resulting sequences were then de-duplicated. Subsequently, the virus sequences have been annotated using Diamond software (version 0.9.21.122) and the National Center for Biotechnology Information (NCBI) Non-Redundant Protein database.^1^

### Confirmation of virus-like contigs

To confirm the detection of viral-like contigs in the strains tested, the presence of each contig was confirmed by RT-PCR using the appropriate primer pairs. The RNA samples were used as RT-PCR templates and specific DNA bands were amplified using the Vazyme HiScript- II One Step qRT-PCR SYBR- Green Kit (no. #Q221-01, Nanjing, China) using the corresponding primers listed in the Supplementary Table S1. DsRNA extraction was also used to confirm the presence of viral-like contigs, as previously described (Li et al., 2015).

### Phylogenetic analysis

Open reading frames (ORFs) were determined with the aid of the ORF Finder program from NCBI. Then, to determine the reliability of a given branching pattern, phylogenetic trees were constructed from RNA-dependent RNA polymerase (RdRp) sequences using the neighbor-joining (NJ) method and tested with 1,000 bootstrap replicates in the MEGA 11 software. Multiple sequence alignments were performed by ClustalW. Accession numbers of the species for building the evolutionary tree are listed in the Supplementary Table S2.

## Result

### Viral sequences in the metatranscriptome of Foc

**Figure 1:**
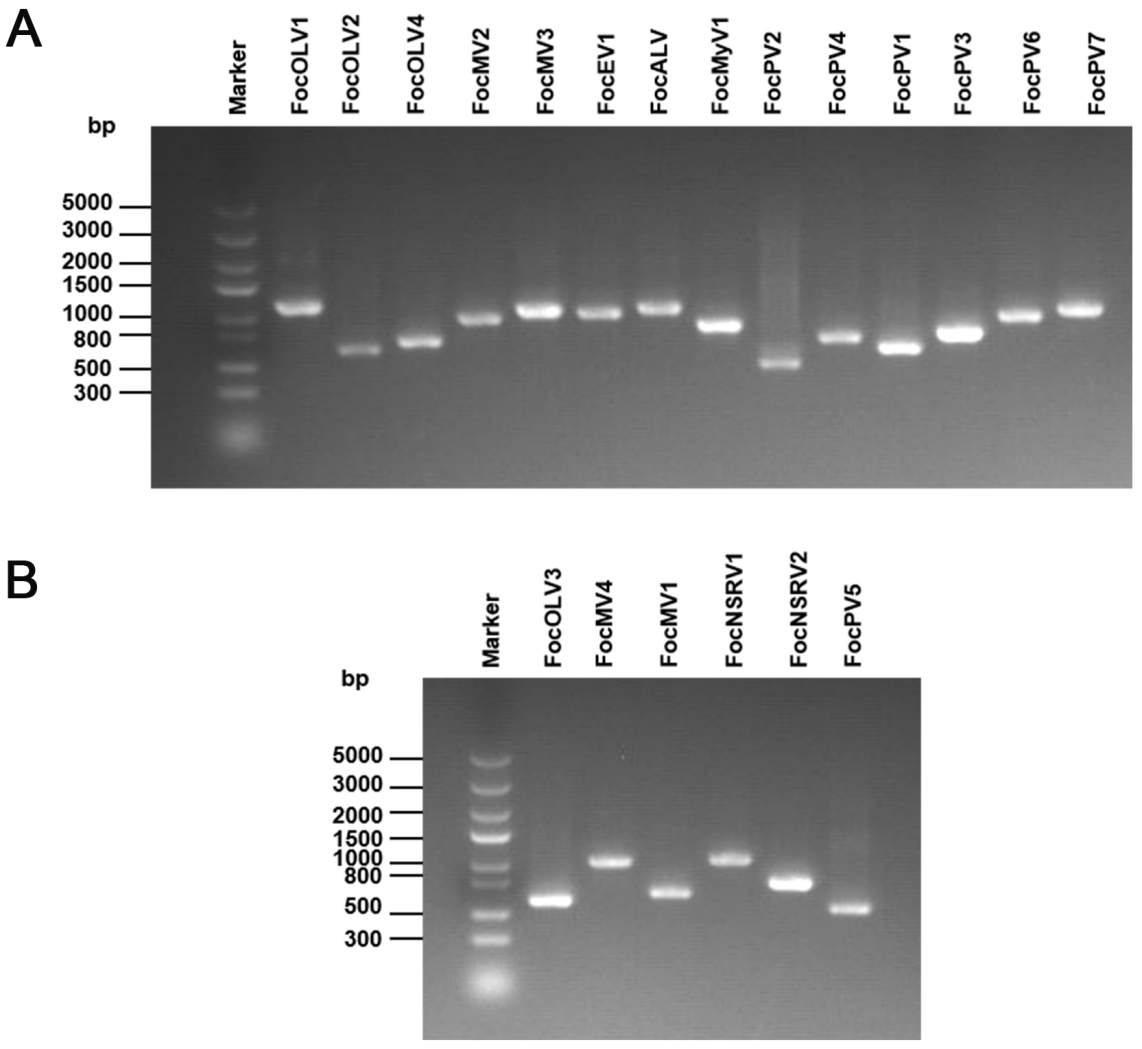
RT-PCR confirmation of 20 viral-like contigs in Foc. **(A)** RT-PCR confirmation of contigs in the RNA samples of group 1. **(B)** RT-PCR confirmation of contigs in the RNA samples of group 2. The viral primers were designed according to the contig sequences. Primers used and predicted sizes of PCR products are listed in Supplementary Table S1. Lane M, Trans5K DNA Marker (Transgen, China); Lane 1 to 20, abbreviates of viruses (see Table 1).

A collection of 246 isolates (group 1 of 146 and group 2 of 100) of Foc isolated from banana samples was used. Among 246 Foc isolates, only 20 in group 1 are Foc1, while all other isolates in two groups are Foc4. A total of 178,543,328 raw reads were generated by Illumina NGS. The assembly of the high-quality reads (QS ≥ 20) yielded about 287,540 contigs (Supplementary Table S3). All contigs were subjected to BLAST analysis. In group 1, partial or nearly complete genome segments of 162 viruses were obtained from the analysis (Supplementary Table S4). Similarly, 70 viruses were obtained in group 2 (Supplementary Table S5). Viruses from different pools with >95% identity at the amino acid level were considered variants of the same virus; the sequence from each virus with the highest read coverage and sequence length was selected as representative after manual inspection of the alignment. Since low coverage and short contigs are known to be prone to error, we focus on further analyzing contigs >800 bp and high coverage contigs for RT-PCR verification. In particular, we focused on these contigs which were identified as viruses potentially related to fungi. Combined with RT-PCR amplification results, the screening identified 20 virus-related sequences from different viral families (Figure 1; Table 1). Further, we confirmed the presence of five contigs (contig9, contig5527, contig6366, contig16483, and contig20141) in each specific strain by the dsRNA extraction (Supplementary Figure S1). Based on sequence analysis, viruses were classified into different taxonomical groups, including 10 viruses that were predicted to represent +ssRNA viruses belonging to three different viral families: *Botourmiaviridae*, *Mitoviridae*, and *Endornaviridae*. The genomes of two -ssRNA viruses were associated with those of viruses in the family *Mymonaviridae* and a novel, unclassified family of the order *Bunyavirales*, respectively. The remaining dsRNA genomes were related to those of members of the family *Partitiviridae*. There is one additional virus, probably representing a member of an unassigned fungal alphavirus-like group.

**Table 1:** Assembled sequences with similarity to previously described viruses.

Number	Contig number	GenBank accession number	Contig length (bp)	Name of putative viruses	Best match^a^	aa identity (%)	Genome type	Family/genus
1	Contig97	OQ685967	2,750	*Fusarium oxysporum* f. sp. *cubense* ourmia-like virus 1 (FocOLV1)	Fusarium mangiferae botourmiavirus 2 (UBZ25887.1)	90	+ssRNA	*Botourmiaviridae*
2	Contig1565	OQ685968	3,253	*Fusarium oxysporum* f. sp. *cubense* ourmia-like virus 2 (FocOLV2)	Botoulivirus sp. (UJQ92030.1)	60	+ssRNA	*Botourmiaviridae*
3	Contig36412	OQ685969	2,766	*Fusarium oxysporum* f. sp. *cubense* ourmia-like virus 4 (FocOLV4)	Botourmiaviridae sp. (WAK77841.1)	85	+ssRNA	*Botourmiaviridae*
4	Contig120	OQ685970	2,444	*Fusarium oxysporum* f. sp. *cubense* ourmia-like virus 3 (FocOLV3)	Fusarium solani ourmia-like virus 1 (WAS28562.1)	49	+ssRNA	*Botourmiaviridae*
5	Contig9	OQ685971	2,471	*Fusarium oxysporum* f. sp. *cubense* mitovirus 2 (FocMV2)	Fusarium andiyazi mitovirus 1 (QPK91779.1)	88	+ssRNA	*Mitoviridae*
6	Contig5103	OQ685972	2,378	*Fusarium oxysporum* f. sp. *cubense* mitovirus 4 (FocMV4)	Colletotrichum fructicola mitovirus 1 (BBN51032.1)	95	+ssRNA	*Mitoviridae*
7	Contig5527	OQ685973	2,388	*Fusarium oxysporum* f. sp. *cubense* mitovirus 1 (FocMV1)	Plasmopara viticola lesion associated mitovirus 7 (WAK72427.1)	93	+ssRNA	*Mitoviridae*
8	Contig20494	OQ685974	2,356	*Fusarium oxysporum* f. sp. *cubense* mitovirus 3 (FocMV3)	Albatrellopsis flettii mitovirus 1 (QUP79368.1)	43	+ssRNA	*Mitoviridae*
9	Contig20141	OQ685975	4,012	*Fusarium oxysporum* f. sp. *cubense* endornavirus 1 (FocEV1)	Agaricus bisporus endornavirus 1 (YP_010086750.1)	25	+ssRNA	*Endornaviridae*
10	Contig2840	OQ685976	1,902	*Fusarium oxysporum* f. sp. *cubense* alphavirus-like virus (FocALV)	Fusarium graminearum alphavirus-like virus 1 (QGW08824.1)	29	+ssRNA	unclassified
11	Contig24	OQ685977	9,485	*Fusarium oxysporum* f. sp. *cubence* mymonavirus 1 (FocMyV1)	Fusarium proliferatum mymonavirus 1 (UWK02084.1)	95	-ssRNA	*Mymonaviridae*
12	Contig14157	OQ685978	10,342	*Fusarium oxysporum* f. sp. *cubense* negative-stranded RNA virus 1 (FocNSRV1)	Sanya Mymon tick virus 1 (UYL95358.1)	99	-ssRNA	*Mymonaviridae*
13	Contig14713	OQ685979	6,712	*Fusarium oxysporum* f. sp. *cubense* negative-stranded RNA virus 2 (FocNSRV2)	Grapevine-associated mycobunya-like virus 4 (QXN75455.1)	57	-ssRNA	unclassified
14	Contig11434	OQ685986	990	*Fusarium oxysporum* f. sp. *cubense* partitivirus 2 (FocPV2)	Partitiviridae sp. (UDL14404.1)	72	dsRNA	*Partitiviridae*
15	Contig6366	OQ685980	1,376	*Fusarium oxysporum* f. sp. *cubense* partitivirus 4 (FocPV4)	Flammulina betapartitivirus 1 (BDV50554.1)	61	dsRNA	*Partitiviridae*
16	Contig75904	OQ685981	1,030	*Fusarium oxysporum* f. sp. *Cubense* partitivirus 1 (FocPV1)	Trichoderma citrinoviride partitivirus 1 (AZT88592.1)	55	dsRNA	*Partitiviridae*
17	Contig16483	OQ685982	1,856	*Fusarium oxysporum* f. sp. *cubense* partitivirus 3 (FocPV3)	Gaeumannomyces tritici partitivirus 2 (AZT88604.1)	67	dsRNA	*Partitiviridae*
18	Contig99419	OQ685983	825	*Fusarium oxysporum* f. sp. *cubense* partitivirus 5 (FocPV5)	Sarcosphaera coronaria partitivirus (QLC36806.1)	54	dsRNA	*Partitiviridae*
19	First_Contig2653	OQ685984	1,909	*Fusarium oxysporum* f. sp. *cubense* partitivirus 6 (FocPV6)	Heterobasidion partitivirus 5 (ADV15444.1)	80	dsRNA	*Partitiviridae*
20	First_Contig2479	OQ685984	1,646	*Fusarium oxysporum* f. sp. *cubense* partitivirus 7 (FocPV7)	Podosphaera prunicola partitivirus 1 (ATS94410.1)	61	dsRNA	*Partitiviridae*

a The Genbank accession number for each virus is listed in the parentheses.

#### *Botourmiaviridae*-related sequences

**Figure 2:**
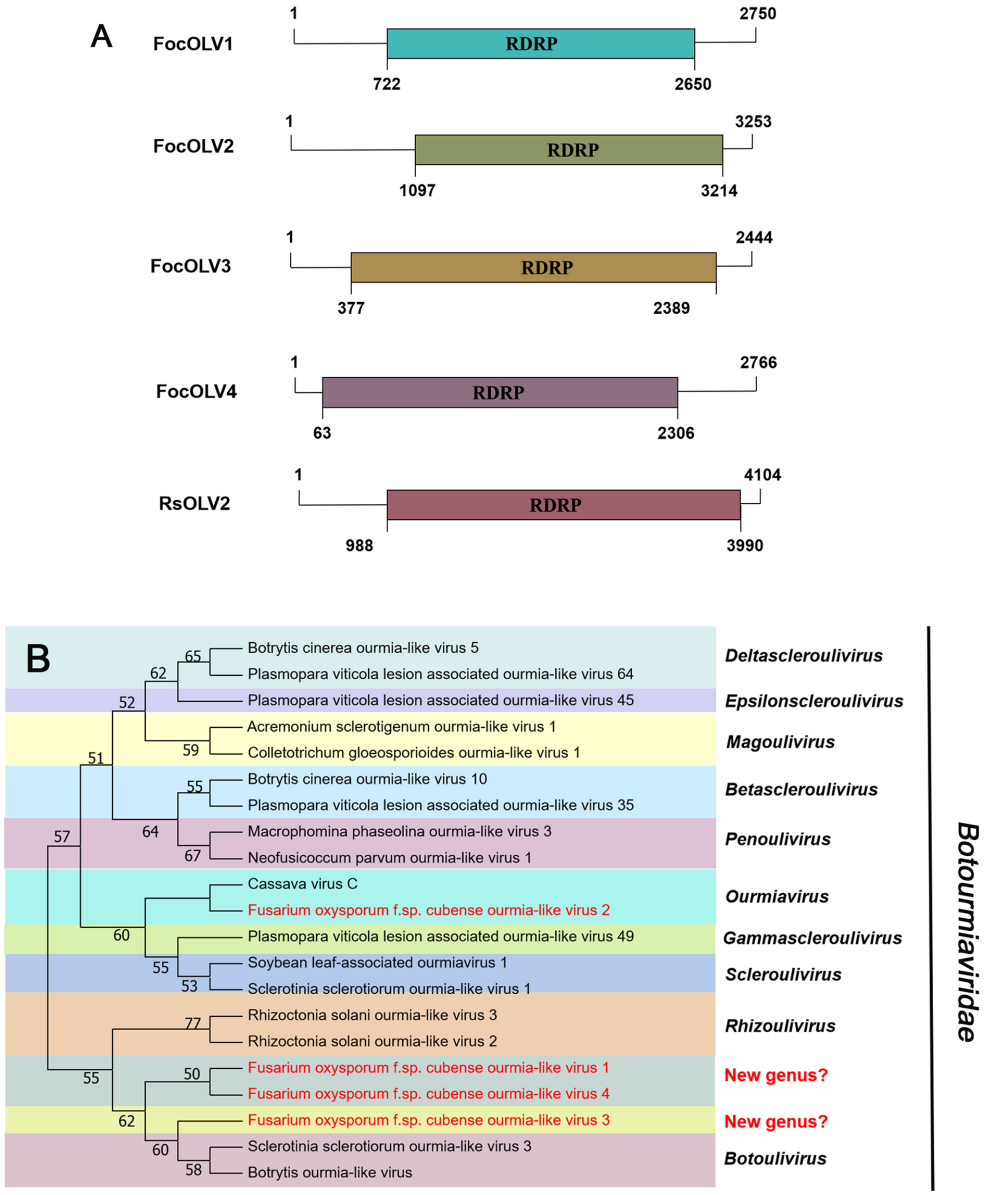
Genome organization and phylogenetic analysis of viruses in the family *Botourmiaviridae*. **(A)** Schematic diagram showing the genome organization of viruses, including Fusarium oxysporum f. sp. cubense ourmia-like virus 1 (FocOLV1), Fusarium oxysporum f. sp. cubense ourmia-like virus 2 (FocOLV2), Fusarium oxysporum f. sp. cubense ourmia-like virus 3 (FocOLV3), Fusarium oxysporum f. sp. cubense ourmia-like virus 4 (FocOLV4), and Rhizoctonia solani ourmia-like virus 2 (RsOLV2). The genomic structure of RsOLV2 was selected as a representative of the family. **(B)** Phylogenetic analysis based on the RdRP of putative viruses in the family *Botourmiaviridae* using the neighbor-joining (NJ) method. Red-marked viruses are isolated in Foc. Viruses without color annotation are representatives of known species of the viral family. Genus clades are circumscribed in coloured blocks, with genus names and the family name shown to the right of the tree. Bootstrap percentage greater than 50% are shown.

The family *Botourmiaviridae* includes 12 genera and viruses with positive-sense RNA genomes that infect plants and fungi. Generally, members of this family, except *Ourmiavirus*, are non-encapsidated fungal viruses with a monopartite and monocistronic genome of 2,000–5,300 nucleotides containing a single ORF encoding the RNA-dependent RNA polymerase (RdRp; Ayllón et al., 2020). Contig97 was 2,750 nt in length, possessing a large ORF encoding a 642 aa peptide showing homology to the RdRp sequence of Fusarium mangiferae botourmiavirus 2, with an aa sequence identity of 90% (Table 1; Figure 2A). We have named it Fusarium oxysporum f. sp. cubense ourmia-like virus 1 (FocOLV1). Contig1565 was 3,253 nt long, encoding a putative RdRp of 705 aa, which was 60% identical to the sequence of *Botoulivirus* sp., and it was named Fusarium oxysporum f. sp. cubense ourmia-like virus 2 (FocOLV2). Contig120 was 2,444 nt in length encoding a putative RdRp of 670 aa, namely Fusarium oxysporum f. sp. cubense ourmia-like virus 3 (FocOLV3). The RdRp of FocOLV3 was most homologous to the RdRp sequence of Magnaporthe oryzae ourmia-like virus (MoOLV) with aa sequence identity of 48%. Contig36412 is 2,766 nt, with a complete ORF encoding for 747 aa. This putative protein showed 85% identity with Phomopsis longicolla RNA virus 1 (PlRV1) RdRP, and we named this virus Fusarium oxysporum f. sp. cubense ourmia-like virus 4 (FocOLV4; Table 1; Figure 2A). To establish the phylogeny of these viruses with other viruses of family *Botourmiaviridae*, a phylogenetic tree was constructed using the neighbor-joining (NJ) method based on the viral RdRp aa sequences (Figure 2B). Results showed that FocOLV2 was mostly related to Cassava virus C, which belongs to the genus *Ourmiavirus*. Specifically, members of the genus *Ourmiavirus* were previously reported to be plant viruses with non-enveloped tri-segmented genome. This is the first report of an ourmiavirus infecting a fungus. FocOLV1 and FocOLV4 are clustered together, and form an independent branch in the evolutionary tree, suggesting that they may belong to a new genus in the family *Botourmiaviridae* (Figure 2B). FocOLV3 also form an independent branch in this phylogenetic tree, implying that it may be a novel genus in the family *Botourmiaviridae*.

#### *Mitoviridae*-related sequences

**Figure 3:**
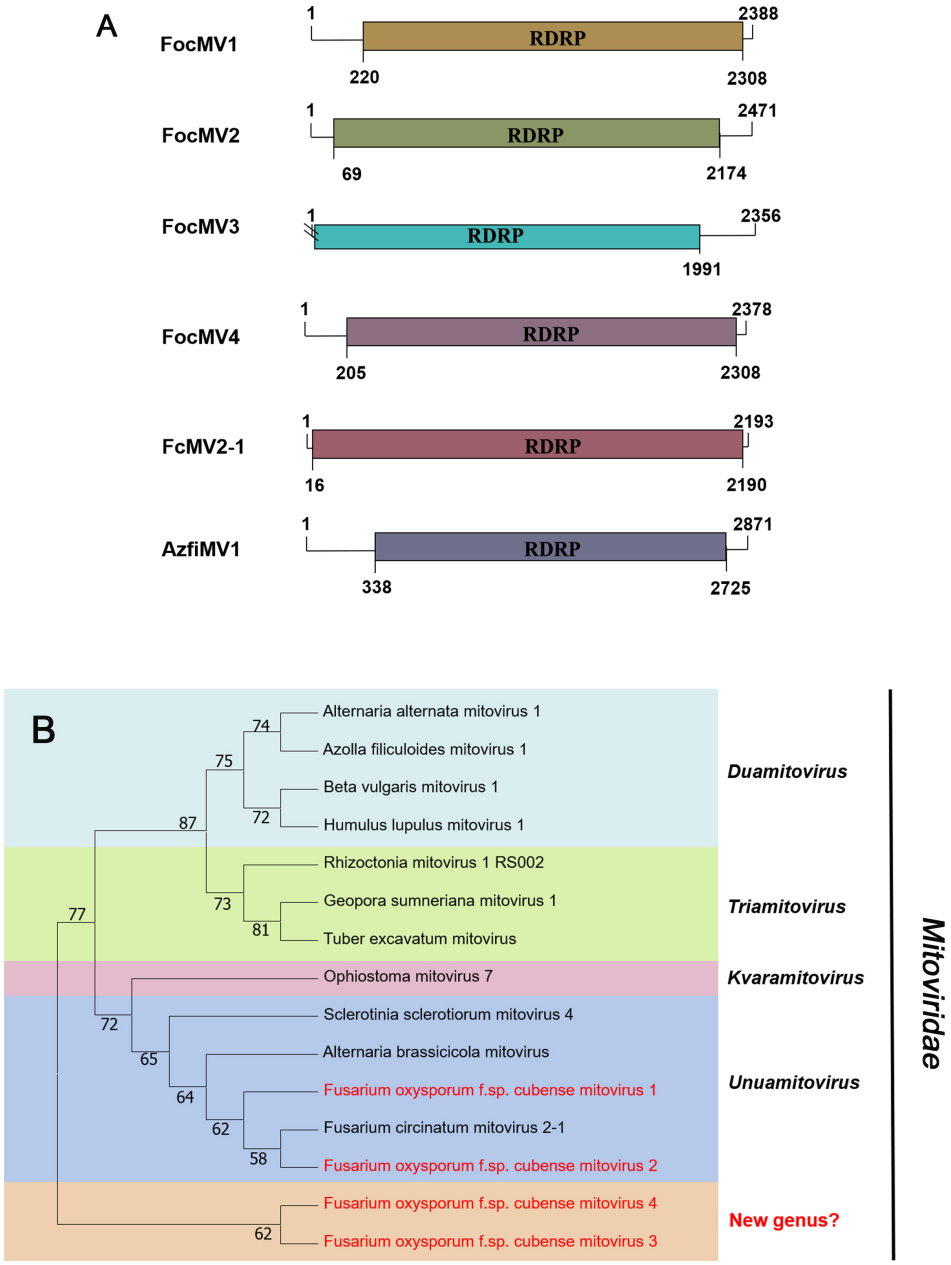
Genome organization and phylogenetic analysis of viruses in the family *Mitoviridae*. **(A)** Schematic diagram showing the genome organization of viruses, including Fusarium oxysporum f. sp. cubense mitovirus 1 (FocMV1), Fusarium oxysporum f. sp. cubense mitovirus 2 (FocMV2), Fusarium oxysporum f. sp. cubense mitovirus 3 (FocMV3), Fusarium oxysporum f. sp. cubense mitovirus 4 (FocMV4), Fusarium circinatum mitovirus 2-1 (FcMV2-1), and Azolla filiculoides mitovirus 1 (AzfiMV1). The genomic structures of FcMV2-1 and AzfiMV1 were selected as representative members of the family. **(B)** Neighbor-joining (NJ) method phylogenetic tree based on the core RNA-dependent RNA polymerase (RdRP) of putative viruses in the family *Mitoviridae*. Red-marked viruses are isolated in Foc. Viruses without color annotation are representatives of known species of the viral family. Genus clades are circumscribed in coloured blocks, with genus names and the family name shown to the right of the tree. Bootstrap percentage greater than 50% are shown.

According to the current ICTV report, members of the family *Mitoviridae* are naked +ssRNA viruses that contain a single ORF encoding the RdRP, with a genome size of 2,151–4,955 nt (Ghabrial and Suzuki, 2009; Jacquat et al., 2023). Four fragments, conting9 (2,471 nt), conting5103 (2,378 nt), conting5527 (2,388 nt), and contig20494 (2,356 nt) were predicted to encode a mitoviral RdRp, namely Fusarium oxysporum f. sp. cubense mitovirus 2 (FocMV2), Fusarium oxysporum f. sp. cubense mitovirus 4 (FocMV4), Fusarium oxysporum f. sp. cubense mitovirus 1 (FocMV1), Fusarium oxysporum f. sp. cubense mitovirus 3 (FocMV3; Table 1; Figure 3A), respectively. The RdRp of FocMV2 was most similar to Fusarium andiyazi mitovirus 1 with an aa sequence identity of 88%, while FocMV4 was most similar to Colletotrichum fructicola mitovirus 1 with an aa sequence identity of 95%. FocMV1 was most similar to Plasmopara viticola lesion associated mitovirus 7, and FocMV3 was most similar to Albatrellopsis flettii mitovirus 1, with an aa sequence identity of 93 and 43%, respectively (Table 1; Figure 3A). Phylogenetic analysis using the RdRp aa sequences showed that FocMV1 and FocMV2 were clustered within the genus *Unuamitovirus* based on the NJ method. Specifically, FocMV3 and FocMV4 are clustered together, but form an independent branch, meaning that they may be members of a new genus (Figure 3B).

#### *Endornaviridae*-related sequences

**Figure 4:**
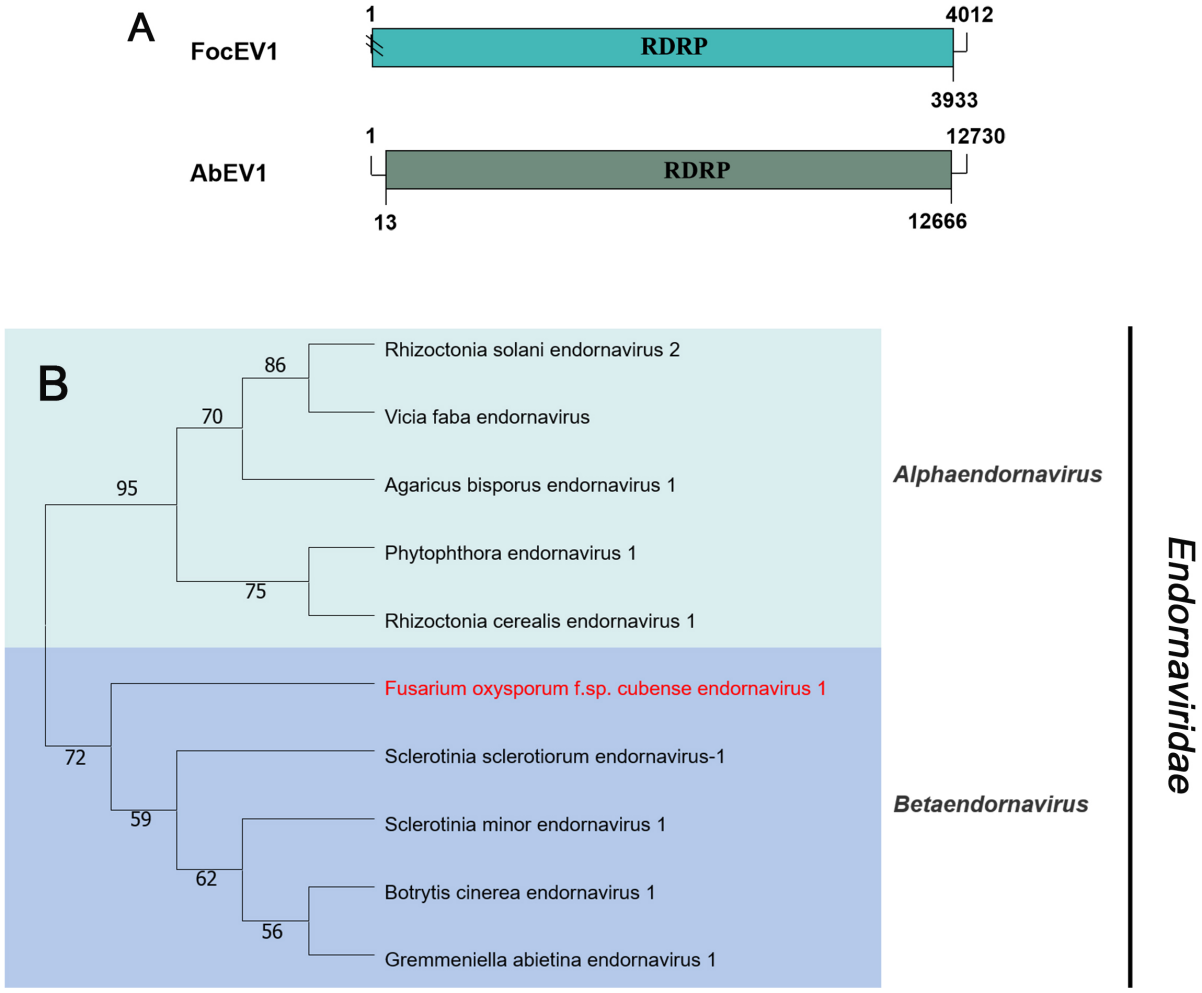
Genome organization and phylogenetic analysis of viruses in the family *Endornaviridae*. **(A)** Schematic diagram showing the genome organization of viruses, containing Fusarium oxysporum f. sp. cubense endornavirus 1 (FocEV1) and Agaricus bisporus endornavirus 1 (AbEV1). The genomic structure of AbEV1 was selected as a representative of the family. **(B)** Neighbor-joining (NJ) method phylogenetic tree based on the core RNA-dependent RNA polymerase (RdRP) of putative viruses in the family *Endornaviridae*. Red-marked viruses are isolated in Foc. Viruses without color annotation are representatives of known species of the viral family. Genus clades are circumscribed in coloured blocks, with genus names and the family name shown to the right of the tree. Bootstrap percentage greater than 50% are shown.

Viruses in the family *Endornaviridae* have linear ssRNA genomes, ranging in length from 10–17 kb, and contain a single large ORF. The family consists of two genera: *Alphaendornavirus* and *Betaendornavirus*. Viruses are divided based on genome size, host, and presence of unique domains (Valverde et al., 2019). According to the BLASTx search, only one fragment (contig20141) of 4,012 nt, contains a large ORF encoding RdRp, showing homology to Agaricus bisporus endornavirus 1 (AbEV1) with 25% identity, namely Fusarium oxysporum f. sp. cubense endornavirus 1 (FocEV1). The phylogenetic analysis showed that FocEV1 is a new member of the genus *Betaendornavirus* (Figure 4).

#### *Mymonaviridae*-related sequences

**Figure 5:**
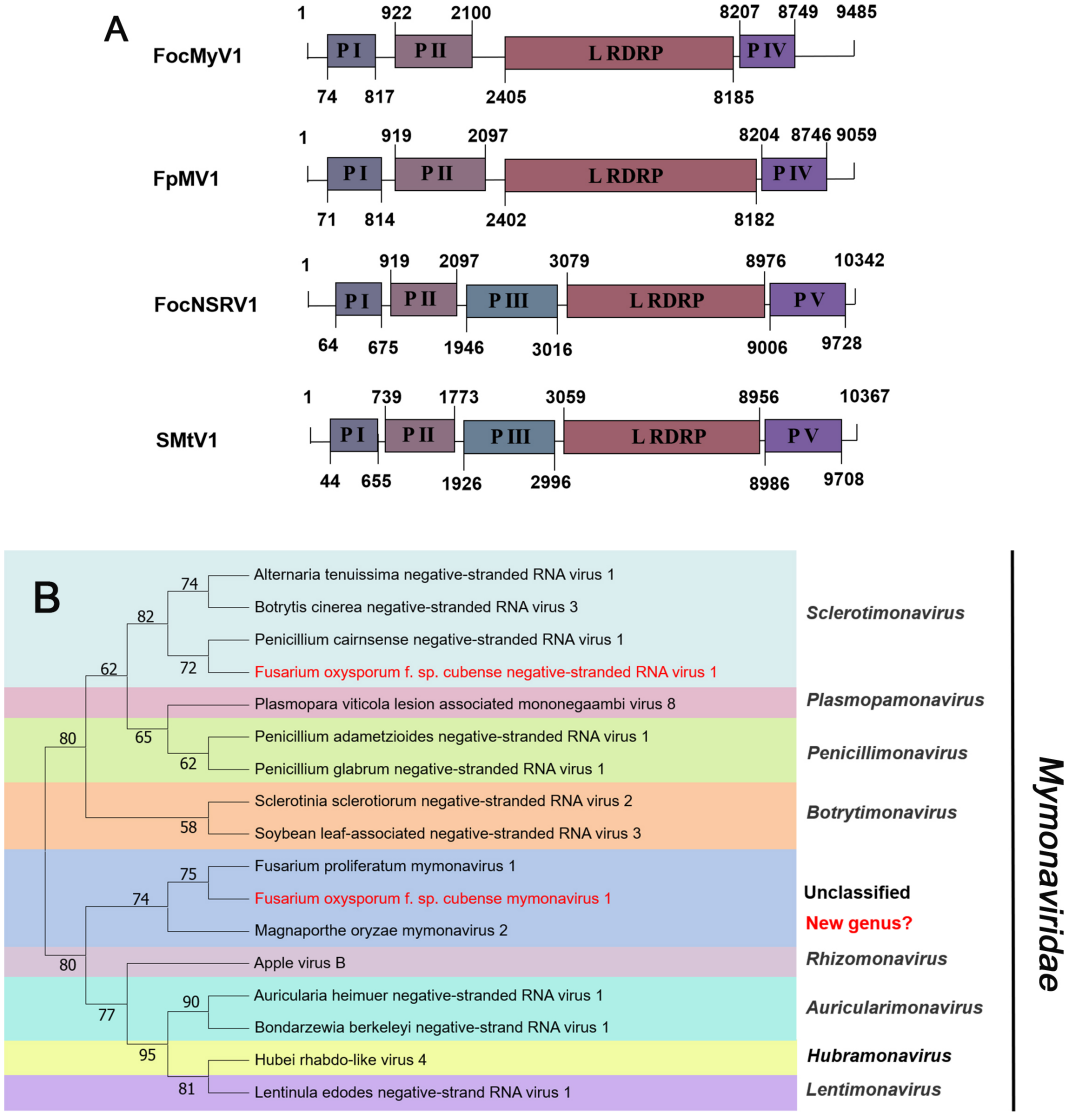
Genome organization and phylogenetic analysis of viruses in the family *Mymonaviridae*. **(A)** Schematic diagram showing the genome organization of viruses, includin Fusarium oxysporum f. sp. cubense mymonavirus 1 (FocMyV1), Fusarium oxysporum f. sp. cubense negative-stranded RNA virus 1 (FocNSRV1), Fusarium proliferatum mymonavirus 1 (FpMV1) and Sanya Mymon tick virus 1 (SMtV1). The genomic structure of FpMV1 and SMtV1 were selected as representatives of the family. **(B)** Neighbor-joining (NJ) method phylogenetic tree based on the core RNA-dependent RNA polymerase (RdRP) of putative viruses in the family *Mymonaviridae*. Red-marked viruses are isolated in Foc. Viruses without color annotation are representatives of known species of the viral family. Genus clades are circumscribed in coloured blocks, with genus names and the family name shown to the right of the tree. Bootstrap percentage greater than 50% are shown.

Negative-sense RNA viruses (NSRVs) are a large and diverse group of viruses. They infect a very wide range of hosts including vertebrates (mainly mammals), invertebrates (mainly arthropods), plants, and fungi. Based on whether their RNA genomes are segmented or not, NSVs are generally classified into two large viral orders: non-segmented negative-sense RNA viruses (nsNSRVs) and segmented negative-sense RNA viruses (sNSRVs). Most nsNSRVs belong to the order *Mononegavirales*, which currently are classified into 11 families (Kuhn et al., 2020). Within the order of *Mononegavirales*, there are two families containing members that have fungi as their primary hosts: *Mymonaviridae* and *Rhabdoviridae* (Kondo et al., 2022). According to the ICTV description, the typical mymonavirus genome is approximately 10 kb, has five or six major non-overlapping ORFs, and has no poly(A) tail structure at the 3′ end (Jiāng et al., 2019). At least one virus in the family *Mymonaviridae* induces hypovirulence in its fungal host, and Sclerotinia sclerotiorum negative-stranded RNA virus 1 is one such virus (Liu et al., 2014). Contig24 was 9,485 nt in length, showing 95% identical to Fusarium proliferatum mymonavirus 1 (FpMV1), a member of an unclassified genus in the family *Mymonaviridae*, using the BLASTx search (Table 1). Thus, we named the virus Fusarium oxysporum f. sp. cubense mymonavirus 1 (FocMyV1). Sequence analysis showed that FocMyV1 has four ORFs, including a large ORF-encoding RdRP, which is similar to FpMV1 (Figure 5A). Contig14157 was 10,342 nt long, with a predicted amino-acid sequence most similar to Sanya Mymon tick virus 1 (SMtV1), with 99% identity. We named it Fusarium oxysporum f. sp. cubense negative-stranded RNA virus (FocNSRV1). Sequence analysis showed that FocNSRV1 has five ORFs, which is similar to SMtV1 (Figure 5A). Phylogenetic analysis based on the RdRP of putative viruses in the family *Mymonaviridae* suggests that FocNRSV1 likely belongs to the genus *Sclerotimonavirus*, while FocMyV1 belongs to an unclassified genus in the family *Mymonaviridae* (Figure 5B).

#### *Partitiviridae*-related sequences

The family *Partitiviridae* contains five genera, *Alphapartitivirus*, *Betapartitivirus*, *Deltapartitivirus*, *Gammapartitivirus*, and *Cryspovirus* that are a widely spread group of viruses infecting plants, fungi, and protozoa. Members of each genus have characteristic hosts: either plants or fungi for genera *Alphapartitivirus* and *Betapartitivirus*, fungi for genus *Gammapartitivirus*, plants for genus *Deltapartitivirus*, and protozoan for genus *Cryspovirus*. Generally, the genome of partitiviruses consists of two dsRNA segments of 1.3 to 2.4 kb each, the larger of which encodes the replicase and the smaller the structural proteins. Nevertheless, additional dsRNA segments may also be present (Vainio et al., 2018).

**Figure 6:**
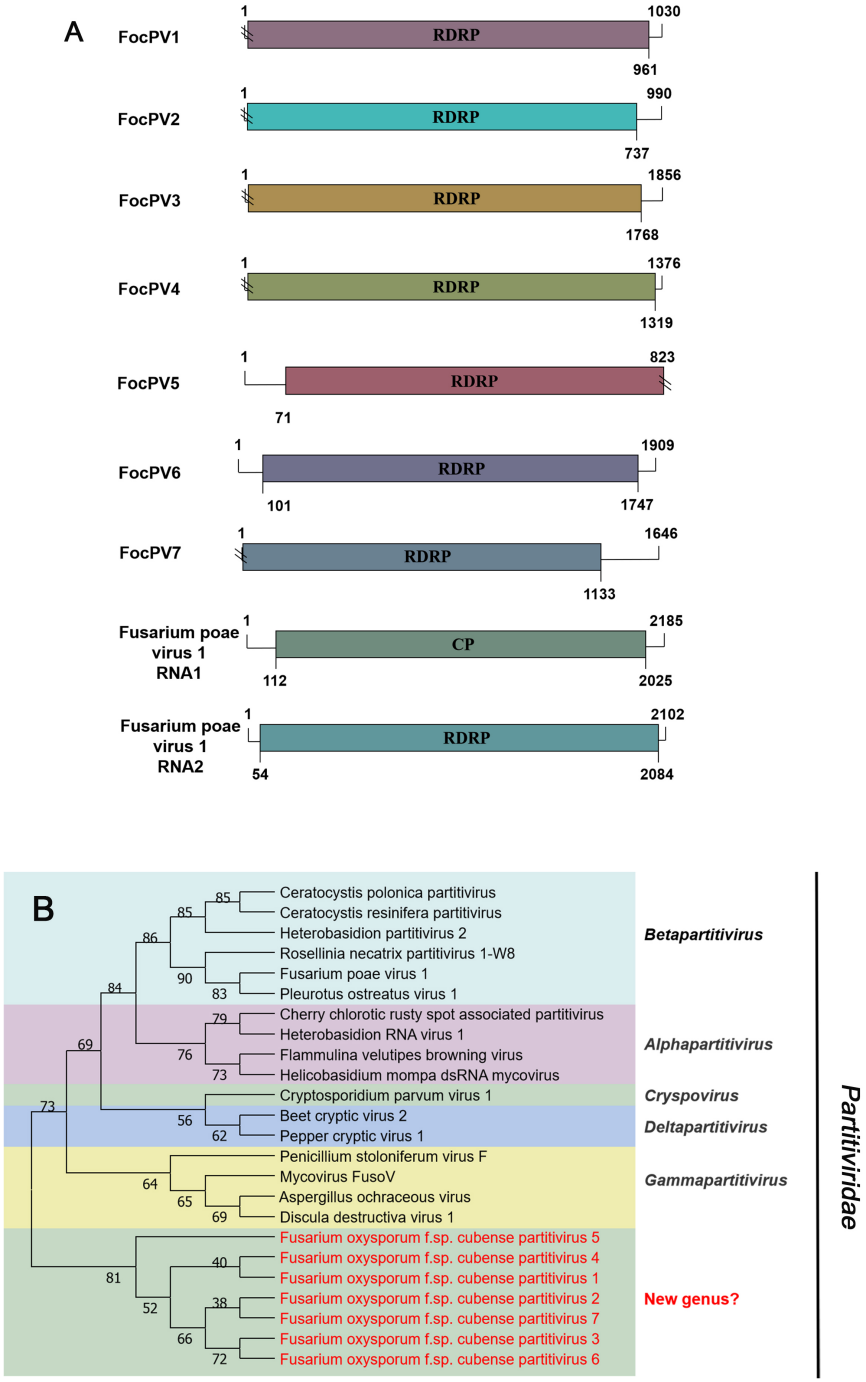
Genome organization and phylogenetic analysis of viruses in the family *Partitiviridae*. **(A)** Schematic diagram showing the genome organization of viruses, including Fusarium oxysporum f. sp. cubense partitivirus 1 (FocPV1), Fusarium oxysporum f. sp. cubense partitivirus 2 (FocPV2), Fusarium oxysporum f. sp. cubense partitivirus 3 (FocPV3), Fusarium oxysporum f. sp. cubense partitivirus 4 (FocPV4), Fusarium oxysporum f. sp. cubense partitivirus 5 (FocPV5), Fusarium oxysporum f. sp. cubense partitivirus 6 (FocPV6), Fusarium oxysporum f. sp. cubense partitivirus 7 (FocPV7) and Fusarium poae virus 1 (FpV1). The genomic structure of FpV1 was selected as representative of the family. **(B)** Neighbor-joining (NJ) method phylogenetic tree based on the core RNA-dependent RNA polymerase (RdRP) of putative viruses in the family *Partitiviridae*. Red-marked viruses are isolated in Foc. Viruses without color annotation are representatives of known species of the virus family. Genus clades are circumscribed in coloured blocks, with genus names and the family name shown to the right of the tree.

Seven sequences related to viruses within the family *Partitiviridae* were identified in Foc. Contig75904, contig11434, contig16483, contig6366, and first-contig2479 contained an incomplete ORF encoding RdRP (Figure 6A), which showed the greatest similarity with the Rosellinia necatrix partitivirus 1-W8 (RnPV1-W8), Fusarium solani partitivirus 2 (FsPV2), Gaeumannomyces tritici partitivirus 2 (GtPV2), Ceratocystis polonica partitivirus (CpPV) and Podosphaera prunicola partitivirus 1 (PpPV1), respectively (Table 1). They all had less than 75% similarity with known viruses. We named these novel partitiviruses as Fusarium oxysporum f. sp. cubense partitivirus 1 (FocPV1), Fusarium oxysporum f. sp. cubense partitivirus 2 (FocPV2), Fusarium oxysporum f. sp. cubense partitivirus 3 (FocPV3), Fusarium oxysporum f. sp. cubense partitivirus 4 (FocPV4), Fusarium oxysporum f. sp. cubense partitivirus 7 (FocPV7), respectively. Contig99419 with 1,815 nt contained a complete ORF that encoded a putative protein with 539 aa, and with 50% identity to the RdRP of Sclerotinia sclerotiorum partitivirus S (SsPVS), and we named this novel virus as Fusarium oxysporum f. sp. cubense partitivirus 5 (FocPV5). The first-contig2653 was 1,909 nt and had a complete ORF encoding putative RdRP with 548 aa (Figure 6A). The amino acid sequence of the predicted ORF product was identical to the RdRp of Heterobasidion partitivirus 5 (HpPV5) with 80% identity, namely Fusarium oxysporum f. sp. cubense partitivirus 6 (FocPV6). The amino-acid sequences of the RdRPs of FocPV1-7 showed that they all had less than 80% similarity with known viruses, and we tentatively speculated that they might represent novel members of the family *Partitiviridae*. FocPVs in the phylogenetic tree formed a separate branch from other known viruses, therefore, they might constitute one new separate genus within the *Partitiviridae* (Figure 6B).

### Unclassified viral sequences

Contig2840 was 1902 nt in length, and contained an incomplete ORF encoding putative polyprotein with 588 aa. Blastx analysis showed that this putative protein was most similar to the RdRp of Fusarium graminearum alphavirus-like virus 1 (FgALV1) with 29% identity (Table 1). FgALV1, a member of a new, unclassified family in the alphavirus-like supergroup (Zhang X. et al., 2020). Thus, contig2840 represented a novel Riboviria virus. We named it Fusarium oxysporum f. sp. cubense alphavirus-like virus (FocALV). Thus, FocALV may also be a novel member of an unassigned fungal alphavirus-like group belonging to the alphavirus-like supergroup.

**Figure 7:**
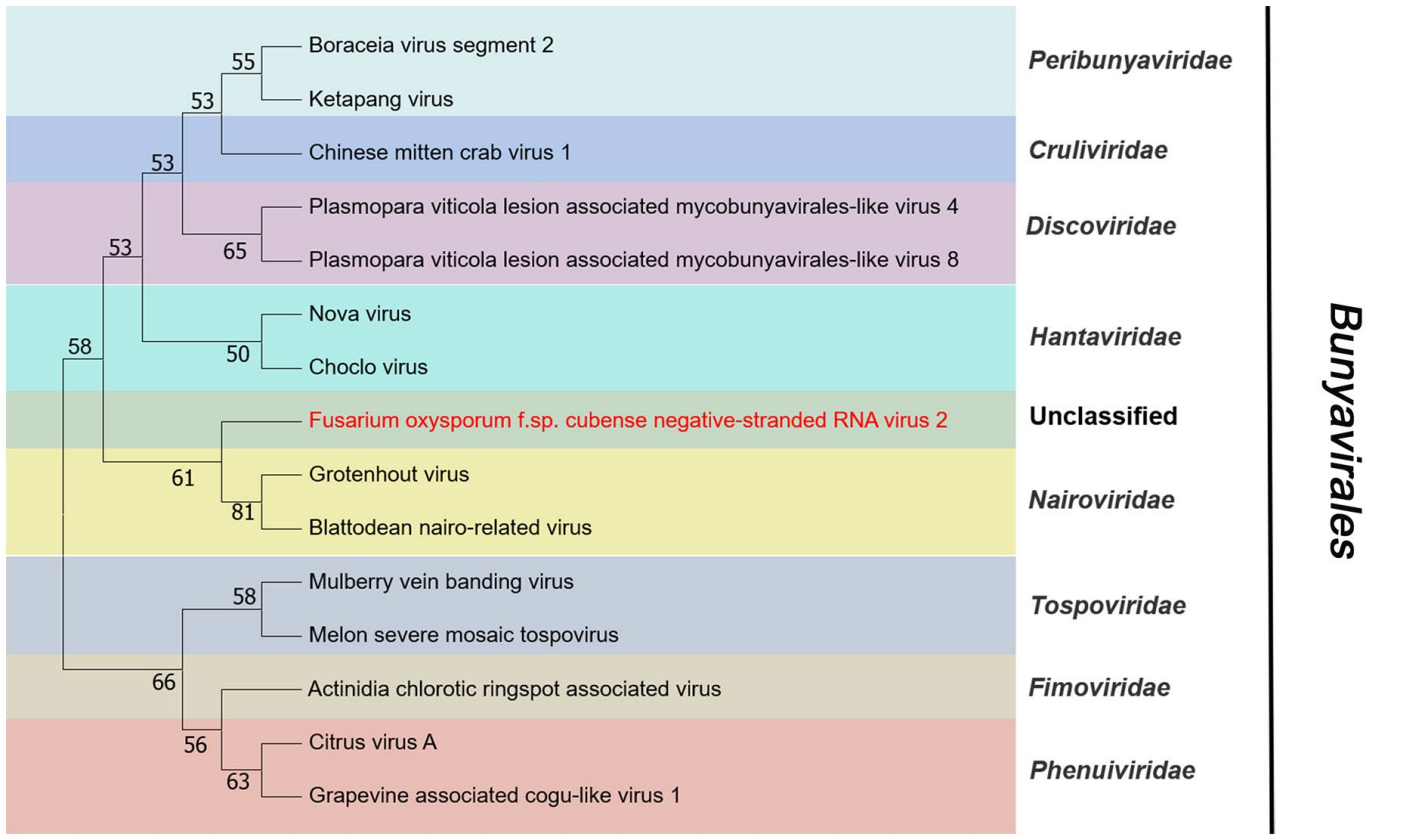
Phylogenetic analysis of viruses in the order *Bunyavirales*. Neighbor-joining (NJ) method phylogenetic tree based on the core RNA-dependent RNA polymerase (RdRP) of putative viruses in the order *Bunyavirales*. Red-marked virus is isolated in Foc. Viruses without color annotation are representatives of known species of the virus family. Family clades are circumscribed in coloured blocks, with family names and the order name shown to the right of the tree. Bootstrap percentage greater than 50% are shown.

Contig14713 was 6,712 nt and contained a complete ORF. It encoded a RdRP with 2,203 aa. The predicted amino acid sequence of this protein was similar to Grapevine-associated mycobunya-like virus 4 (GaMLV4) with 57% identity. GaMLV4 belongs to the unclassified *Bunyavirales*. This suggests that contig14713 likely represents a novel virus of *Bunyavirales*, and we have named it Fusarium oxysporum f. sp. cubense negative-stranded RNA virus 2 (FocNSRV2). Therefore, FocNSRV2 may be a new member of the order *Bunyavirales* (Figure 7).

## Discussion

In this study, a metatranscriptome -based strategy was used to investigate the virome diversity and composition associated with the plant-pathogenic fungus Foc that causes banana Fusarium wilt disease. The aim is to identify the mycoviruses which can influence the pathogenicity of the host to provide potential materials for the biological control of Foc. A total of 246 Foc isolates were collected from different diseased banana plants and the isolates were divided into 2 groups for analysis as described by Ruiz-Padilla et al. (2021) to obtain the most comprehensive overview of the viral profile. Illumina sequencing yielded a total of 287,540 contigs, and bioinformatic analysis assembled 20 mycoviruses with RNA genomes. To our knowledge, this is the first report of the mycoviral diversity in Foc. On the ground of their genomic structures, sequence sequence analyses, and phylogenetic analyses based on RdRp, these mycoviruses in Foc could be tentatively classified into five families (*Botourmiaviridae*, *Mitoviridae*, *Endornaviridae*, *Mymonaviridae*, and *Partitiviridae*) and two non-classified mycoviruses lineages.

Phylogenetic trees are widely used to study evolutionary relationships between species. To assess the phylogenetic relationship of the predicted virus with other members of the viral family, the contig sequences were compared with the RdRp sequences of members of currently known viral genera. Phylogenetic analysis revealed that four botourmiaviruses clustered into three well-supported clades, one belonging to the genus *Ourmiavirus* and others belonging to the new genus. FocOLV1 and FocOLV4 cluster together while FocOLV3 forms a monophyletic clade. Therefore, we propose the creation of two new genera within *Botourmiaviridae*. Similarly, mitoviruses are not clustered in monophyletic lineages and there are two mitoviruses in a separate branch, suggesting the creation of a new genus in the *Mitoviridae*. As shown in Figure 4, FocEV1 is tightly clustered with members of the genus *Betaendornavirus* of the family *Endornaviridae.* The genome of betaendornaviruses is less than 10.7 kb in length and absence of a site-specific nick near the 5′-end of the coding strand, according to the criteria for the classification of the two genera of Endornaviridae in ICTV (Valverde et al., 2019). As FocEV1 has an incomplete genome sequence, whether FocEV1 should be classified as a new member of the genus *Betaendornavirus* requires further work.

In 2014, the first -ssRNA mycovirus was identified in *Sclerotinia sclerotiorum*, leading to the establishment of a new family, *Mymonaviridae* (Liu et al., 2014; Jiāng et al., 2019). As described by ICTV, Sclerotinia sclerotiorum negative stranded RNA virus 1 (SsNSRV-1), a typical member of the *Mymonaviridae*, has six major non-overlapping ORFs encoding six proteins (p I, NP, p III, p IV, L protein, and p VI; Jiāng et al., 2019). Other mymonavirus genomes contain four to seven ORFs, such as Botrytis cinerea negative-stranded RNA virus 7 (BcNSRV7), Húběi rhabdo-like virus 4 (HbRLV-4), and Lentinula edodes negative-strand RNA virus 1 (LeNSRV1; Donaire et al., 2016; Shi et al., 2016; Lin et al., 2019). Similarly, FocMyV1 contains four ORFs and ORF3 encodes the L protein, while FocNSRV1 contains five ORFs and ORF4 encodes the L protein. Moreover, the phylogenetic analysis showed that FocNSRV1 formed an independent clade of *Sclerotimonavirus* in the family *Mymonaviridae.* FocMyV1 formed a tight clustered with Fusarium proliferatum mymonavirus 1 and Magnaporthe oryzae mymonavirus 2. These three viruses formed a separated branch distant from other viruses, implying thatthey represent a new genus in the family *Mymonaviridae.* In brief, we characterized two novel mymonavirus, FocMyV1 and FocNSRV1, in the genus *Sclerotimonavirus* and a new genus, respectively. Most notably, Partitiviride-related viruses found in Foc appear to be phylogenetically on a separate branch.

Similar to other plant pathogenic fungi, mycoviruses in the Lenarviricota phylum were prevalent in Foc isolates (Chiapello et al., 2020; Ruiz-Padilla et al., 2021). Among them were 4 viruses that belong to the family *Botourmiaviridae* and 4 viruses that belong to the family *Mitoviridae*, both of which belong to the phylum Lenarviricota. Due to their mitochondrial localization, mitoviruses are conjecturably unaffected by host antiviral RNA silencing, which may contribute to their prevalence (Shahi et al., 2019). Multiple lines of evidence suggest that mycovirus have been transferred between fungi and plants due to fungus-mediated horizontal gene transfer (HGT) events and long-term co-evolution (Nerva et al., 2017; Abdoulaye et al., 2021). Indeed, there have been reports of horizontal transfer of mitoviruses from fungi to plants, as in the case of Botrytis cinerea mitovirus 10 (BcMV10), which was transmitted from *Botrytis cinerea* to cucumber plants (Wang Q. et al., 2022). Some viruses in the *Mitoviridae* family cause swelling and malformation of host mitochondria, reducing the growth and virulence of the fungus, such as Botrytis cinerea mitovirus 1 (BcMV1), Sclerotinia sclerotiorum mitovirus 1 (SsMV1/HC025) and Sclerotinia sclerotiorum mitovirus 2/KL-1 (SsMV2/KL-1; Wu et al., 2010; Khalifa and Pearson, 2013; Xu et al., 2015). The FocMV2 was most similar to Fusarium circinatum mitovirus 2–1 (FcMV2-1), with a sequence identity of 88% (Table 1). Vitro studies showed that both mycelial growth and spore germination were significantly reduced by the presence of the mitovirus FcMV2-1 (Romeralo et al., 2018). Therefore, whether or not FocMV2 plays a significant role in Foc remains to be explored.

With the application of metatranscriptomic sequencing technology in mycoviral diversity research, a great number of novel mycoviruses have been discovered and identified; and this has supported the progress of research in their related fields, such as virus pathogenesis, the control of related diseases. Further, the mining of rich mycoviral diversity could also be used to explore the interaction mechanism between mycovirus and its host fungus. Although most mycoviruses are cryptic and cause no visible abnormal symptoms in their fungal hosts, they may play an important role in their population biology. To date, at least nine mycoviruses with complete genomic sequences have been reported in *F. oxysporum*, including Fusarium oxysporum chrysovirus 1 (family *Chrysoviridae*), Fusarium oxysporum f. sp. dianthi mycovirus 1 (family *Chrysoviridae*), Fusarium oxysporum alternavirus 1 (family *Alternaviridae*), Fusarium oxysporum mitovirus 1 (family *Mitoviridae*), Fusarium oxysporum f. sp. dianthi mitovirus 1 (family *Mitoviridae*), Fusarium oxysporum f. sp. dianthi hypovirus 2 (family *Hypoviridae*), Fusarium oxysporum ourmia-like virus 1 (family *Botourmiaviridae*), Hadaka Virus 1 (family *Polymycoviridae*), and Fusarium oxysporum mymonavirus 1 (family *Mymonaviridae*; Sharzei et al., 2007; Lemus-Minor et al., 2015; Sato et al., 2020; Torres-Trenas et al., 2020; Torres-Trenas and Pérez-Artés, 2020; Zhao et al., 2020; Wang et al., 2021; Wen et al., 2021; Wang J. et al., 2022). Of these reported mycoviruses, Fusarium oxysporum f. sp. dianthi virus 1 (FodV1), Fusarium oxysporum ourmia-like virus 1 (FoOuLV1) and FoMyV1 are capable of causing hypovirulence of host and can be used as biological control agents (Lemus-Minor et al., 2019; Zhao et al., 2020; Wang J. et al., 2022). This study is the first report of seven partitiviruses from *F. oxysporum*. In fungi, partitiviruses are transmitted intracellularly during cell division, hyphal anastomosis, and sporogenesis (Nibert et al., 2014; Vainio et al., 2018). Normally, partitiviruses tend to infect host fungi asymptomatically, but some viruses significantly affect the morphology and virulence of their hosts and are therefore considered to be a potential source of biocontrol agents against pathogenic fungi (Wang et al., 2014; Xiao et al., 2014; Wang R. et al., 2022). Currently, there are less effective, environmentally friendly and sustainable control methods for Foc (Ploetz, 2015). Therefore, it is urgent that new mycoviruses that significantly reduce host virulence should be exploited as alternative biological control agents of banana Fusarium wilt caused by Foc. In conclusion, this is the first study to show the existence of various mycoviruses in Foc. This pioneering information will be a key for investigating the intricate interactions between mycovirus and Foc and will provide extensive insights into the potential use of mycoviruses as biocontrol agents.

## Data availability statement

The original contributions presented in the study are included in the article/Supplementary material, further inquiries can be directed to the corresponding authors.

## Author contributions

YY executed the experiments and drafted the manuscript. HL and PL designed the research and revised the manuscript. YL, XW, and YZ performed the data and bioinformatics analyses. All authors contributed to the article and approved the submitted version.

## Funding

The work was supported by the National Natural Science Foundation of China (32202381), China Agriculture Research System of MOF and MARA (CARS-31), and Guangdong Basic and Applied Basic Research Foundation (2022A1515140114).

## Conflict of interest

The authors declare that the research was conducted in the absence of any commercial or financial relationships that could be construed as a potential conflict of interest.

## Publisher’s note

All claims expressed in this article are solely those of the authors and do not necessarily represent those of their affiliated organizations, or those of the publisher, the editors and the reviewers. Any product that may be evaluated in this article, or claim that may be made by its manufacturer, is not guaranteed or endorsed by the publisher.

## Supplementary material

The Supplementary material for this article can be found online at: https://www.frontiersin.org/articles/10.3389/fmicb.2023.1193714/full#supplementary-material

## Footnotes

^1^ https://www.ncbi.nlm.nih.gov/
