# Supplementary material for: Metatranscriptome-based strategy reveals the existence of novel mycoviruses in the plant pathogenic fungus Fusarium oxysporum f. sp. cubense
Source: Front Microbiol. 2023 May 18;14:1193714. doi: 10.3389/fmicb.2023.1193714 (PMC10234264; doi:10.3389/fmicb.2023.1193714)
Supplement: Supplementary file 1 [file Data_Sheet_1.zip › Tables 1-3.docx]

Supplementary Material

Metatranscriptome-based strategy reveals the existence of novel mycoviruses in the plant pathogenic fungus *Fusarium oxysporum* f. sp. *cubense*

Yiting Ye, Yingying Liu, Yifei Zhang, Xin Wang, Huaping Li^*^, Pengfei Li^*^

*** Correspondence:**  lipengfei@scau.edu.cn

[huaping@scau.edu.cn](mailto:huaping@scau.edu.cn)

Table S1 List of PCR primers used for viral contig detection

| **Contig number** | **Primer Forward (5’-3’)** | **Primer Reverse (5’-3’)** | **Product Size (bp)** |
| --- | --- | --- | --- |
| Contig97 | TAATACCAAGCCACCGTTGCG | TCATTATTAACGGCGACGATA | 1116 |
| Contig1565 | GGAGTCGTATACCTGACTGTT | CTTGCCAGCCCGCGTTCGTTA | 587 |
| Contig36412 | TGCGGATCTTCCGATCCGACT | ATCCGTCGACTATACGTCGGC | 680 |
| Contig9 | CCTGGATTACTCATCTATCAC | TCATCTTTTTGCAAATTCATA | 925 |
| Contig20494 | GTCATAGTAAATTGGTGGCTG | GGTTATAGGTGGCCCTCTTTT | 1053 |
| Contig20141 | TTCGTCTCGGAATCTCACTTG | CCAGGTGACACCACAATTGCA | 990 |
| Contig2840 | GAGATAATGCTCGACGAAGGT | CGTCTTTGGCGAGGAAGCTGC | 1060 |
| Contig24 | CGGATGGACAGGACGATGATG | CGGATACGCCGGACGAGTCGA | 848 |
| Contig11434 | CCATTGCCATAAGTTCTCGAT | CAAATATCGCGATCCTCAATT | 466 |
| Contig6366 | GGTGAGTTGTGTAGTCATAGT | ACCCCCGATCTTGACCCCAGT | 709 |
| Contig75904 | GTGCATAGGTGTAGACTGTGT | TCAGTTTCATACTGAAGAAGA | 608 |
| Contig16483 | GAGGTGGTTGCGAATTTCAGA | TCATGGTACGTCAAAAACGGC | 780 |
| Contig14157 | TCGGGACTACGCGAAACAGAC | CACGAGCTTCGACAACAATGT | 948 |
| First_Contig2479 | AACATCCCATTGCAGCAGCGG | CCAGAAACAAAGCGAAGGTGA | 1058 |
| Contig120 | GAATCGAACCACAGCCTTAAC | CACCTATCTCGAGTTGCCGAA | 585 |
| Contig5103 | GGACTTTGGACATCATTCCAA | GCTCAGCCACTGATAGATTCC | 1020 |
| Contig5527 | CCTCAGGTAGATACATCTAAA | TACACTCAGGATCTTTAACAA | 664 |
| Contig99419 | GAACATCCCATAGCAGCGGTT | GAAGACTCTTCCGTCGAATGG | 527 |
| Contig14713 | AAGAACAGGATGGAGAACCCC | CTCTCGAACCTCTCTGGCAAG | 785 |
| First_Contig2653 | GGTTGGGTTCGTAGCCTTGGT | ATCAAAAGTGGTTTCACGGAT | 1051 |

Table S2 Accession numbers of the viral species for building the evolutionary tree

| **Species** | **Accession numbers** |
| --- | --- |
| Sclerotinia sclerotiorum ourmia-like virus 3 | MF444276.1 |
| Botrytis cinerea ourmia-like virus 10 | MN605476.1 |
| Plasmopara viticola lesion associated ourmia-like virus 35 | ON813047.1 |
| Botrytis cinerea ourmia-like virus 5 | OL602098.1 |
| Plasmopara viticola lesion associated ourmia-like virus 64 | MN532651.1 |
| Plasmopara viticola lesion associated ourmia-like virus 45 | MN532632.1 |
| Plasmopara viticola lesion associated ourmia-like virus 49 | MN532636.1 |
| Acremonium sclerotigenum ourmia-like virus 1 | ON812991.1 |
| Colletotrichum gloeosporioides ourmia-like virus 1 | MK542706.1 |
| Cassava virus C | NC_013113.1 |
| Macrophomina phaseolina ourmia-like virus 3 | MT062431.1 |
| Neofusicoccum parvum ourmia-like virus 1 | MK584837.1 |
| Rhizoctonia solani ourmia-like virus 2 | MK372906.1 |
| Soybean leaf-associated ourmiavirus 1 | ON812900.1 |
| Humulus lupulus mitovirus 1 | BK010431.1 |
| Beta vulgaris mitovirus 1 | ON605610.1 |
| Azolla filiculoides mitovirus 1 | BK010423.1 |
| Alternaria alternata mitovirus 1 | MK584829.1 |
| Tuber excavatum mitovirus | JN222389.1 |
| Rhizoctonia mitovirus 1 RS002 | KC792591.1 |
| Geopora sumneriana mitovirus 1 | MN043682.1 |
| Ophiostoma mitovirus 7 | KF031943.1 |
| Sclerotinia sclerotiorum endornavirus-1 | NC_021706.1 |
| Sclerotinia minor endornavirus 1 | NC_040631.1 |
| Gremmeniella abietina endornavirus 1 | NC_007920.1 |
| Botrytis cinerea endornavirus 1 | NC_031752.1 |
| Vicia faba endornavirus | NC_007648.1 |
| Rhizoctonia solani endornavirus 2 | NC_055462.1 |
| Rhizoctonia cerealis endornavirus 1 | NC_022619.1 |
| Phytophthora endornavirus 1 | NC_007069.1 |
| Fusarium proliferatum mymonavirus 1 | OK524200.1 |
| Penicillium cairnsense negative-stranded RNA virus 1 | MK584851.1 |
| Botrytis cinerea negative-stranded RNA virus 3 | MN617150.1 |
| Alternaria tenuissima negative-stranded RNA virus 1 | MK584852.1 |
| Apple virus B | MN386971.1 |
| Penicillium glabrum negative-stranded RNA virus 1 | MK584853.1 |
| Penicillium adametzioides negative-stranded RNA virus 1 | MK584858.1 |
| Lentinula edodes negative-strand RNA virus 1 | LC466007.1 |
| Hubei rhabdo-like virus 4 | NC_032783.1 |
| Soybean leaf-associated negative-stranded RNA virus 3 | KT598228.1 |
| Sclerotinia sclerotiorum negative-stranded RNA virus 2 | KP900931.1 |
| Bondarzewia berkeleyi negative-strand RNA virus 1 | T447187.1 |
| Auricularia heimuer negative-stranded RNA virus 1 | MT259204.1 |
| Mycovirus FusoV | NC_003885.1 |
| Discula destructiva virus 1 | NC_002797.1 |
| Aspergillus ochraceous virus | MG887765.1 |
| Pepper cryptic virus 1 | KY923701.1 |
| Beet cryptic virus 2 | NC_038846.1 |
| Cryptosporidium parvum virus 1 | MH311949.1 |
| Rosellinia necatrix partitivirus 1-W8 | AB113347.1 |
| Pleurotus ostreatus virus 1 | NC_006961.1 |
| Heterobasidion partitivirus 2 | NC_038839.1 |
| Fusarium poae virus 1 | MH665658.1 |
| Ceratocystis resinifera partitivirus RNA 1 | NC_010755.1 |
| Ceratocystis polonica partitivirus | AY260756.1 |
| Heterobasidion RNA virus 1 | NC_038827.1 |
| Helicobasidium mompa dsRNA mycovirus | NC_043392.1 |
| Flammulina velutipes browning virus | NC_038826.1 |
| Cherry chlorotic rusty spot associated partitivirus | AJ781401.1 |
| Boraceia virus | MK896611.1 |
| Ketapang virus | MK896548.1 |
| Chinese mitten crab virus 1 | MH717874.1 |
| Plasmopara viticola lesion associated mycobunyavirales-like virus 4 | MN548097.1 |
| Plasmopara viticola lesion associated mycobunyavirales-like virus 8 | MN585278.1 |
| Nova virus | KY780088.1 |
| Choclo virus | KT983773.1 |
| Grotenhout virus | KY700684.2 |
| Blattodean nairo-related virus | MT153554.1 |
| Mulberry vein banding virus | MK681486.1 |
| Melon severe mosaic tospovirus | NC_033834.1 |
| Actinidia chlorotic ringspot associated virus | NC_038769.1 |
| Citrus virus A | OK564750.1 |
| Grapevine associated cogu-like virus 1 | MN520753.1 |

Table S3 Summary statistics of the sequencing data.

| **Group** | **Raw reads** | **Clean reads** | **Clean ratio (Clean reads/raw reads)** | **Q20 ratio (%)** | **Contigs** |
| --- | --- | --- | --- | --- | --- |
| 1 | 89,814,352 | 88,986,886 | 99.08% | 98.30 | 199,623 |
| 2 | 88,728,976 | 87,916,456 | 99.09% | 97.59 | 87,917 |
