# Supplementary material for: Metatranscriptome-based strategy reveals the existence of novel mycoviruses in the plant pathogenic fungus Fusarium oxysporum f. sp. cubense
Source: Front Microbiol. 2023 May 18;14:1193714. doi: 10.3389/fmicb.2023.1193714 (PMC10234264; doi:10.3389/fmicb.2023.1193714)
Supplement: Supplementary file 1 [file Data_Sheet_1.zip › Image 1 figure legend.DOCX]

Fig. S1 Confirmation of the existence of five contigs (contig9, contig5527, contig6366, contig16483, and contig20141) in specific strains by the dsRNA extraction. dsRNA was fractionated on a 1% agarose gel and stained with ethidium bromide. Lane M, DNA marker (DL 15,000 DNA Marker, TaKaRa). Strains BJ14，BY22, BY27, PB13, and BY3, contained viral-like contigs: contig20141, contig9, contig5527, contig6366, and contig16483, respectively.
