# Supplementary figures and images for: Metatranscriptome-based strategy reveals the existence of novel mycoviruses in the plant pathogenic fungus Fusarium oxysporum f. sp. cubense
Source: Front Microbiol. 2023 May 18;14:1193714. doi: 10.3389/fmicb.2023.1193714 (PMC10234264; doi:10.3389/fmicb.2023.1193714)

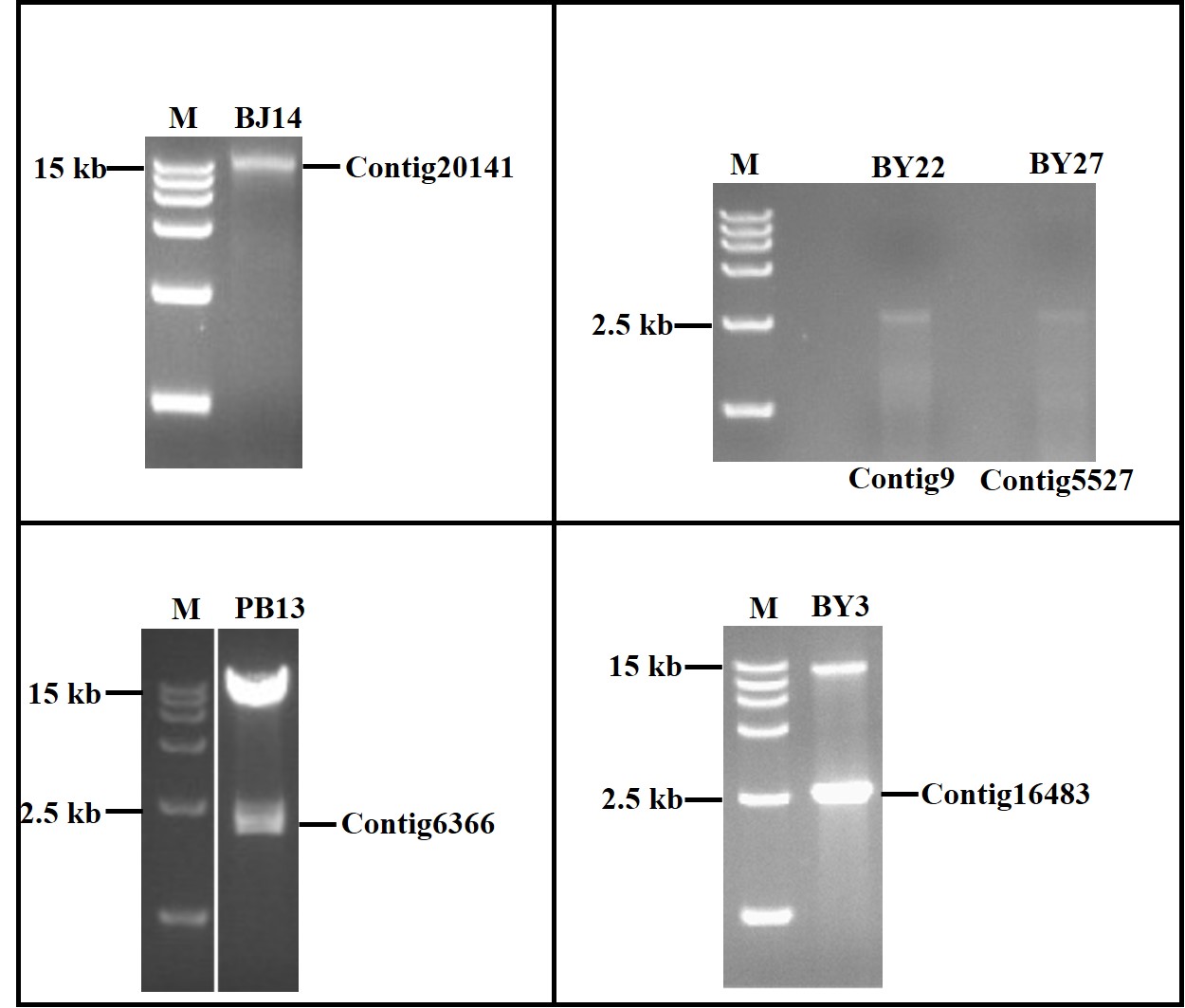

Supplement: Supplementary file 1 [file Data_Sheet_1.zip › Image 1.JPEG]
